# Mechanism Underlying *Bacillus subtilis* BS-Z15 Metabolite-Induced Prevention of Grain Contamination by *Aspergillus flavus*

**DOI:** 10.3390/toxins15120667

**Published:** 2023-11-22

**Authors:** Jingjing Zhao, Jun Yang, Haoran Li, Huanchen Ning, Jiayi Chen, Zhihui Chen, Heping Zhao, Huixin Zhao

**Affiliations:** 1 Xinjiang Key Laboratory of Special Species Conservation and Regulatory Biology, College of Life Science, Xinjiang Normal University, Urumqi 830054, China; zjj980709@163.com (J.Z.); yjun2521@163.com (J.Y.); lhr19980929@163.com (H.L.); nhc19970102@163.com (H.N.); chenjiayi030313@163.com (J.C.); 2 Xinjiang Technical Institute of Physics and Chemistry, Chinese Academy of Sciences, Urumqi 830011, China; 3 Beijing Key Laboratory of Gene Resource and Molecular Development, College of Life Sciences, Beijing Normal University, Beijing 100875, China; hpzhao@bnu.edu.cn

**Keywords:** *Bacillus subtilis* BS-Z15, *Aspergillus flavus*, Mycosubtilin, transcriptome, *A. flavus* growth

## Abstract

*Aspergillus flavus* can cause mildew in corn, peanuts, and other foods as well as animal feed, which seriously endangers human and livestock health; thus, preventing *A. flavus* contamination is imperative. Previous studies have found that the secondary metabolites of *Bacillus subtilis* BS-Z15 have broad-spectrum-inhibiting fungal activity, further confirming that the main active inhibiting fungal substance is Mycosubtilin (Myco). In this paper, corn and peanuts were treated with 0, 100, and 200 μg/mL BS-Z15 secondary metabolites (BS-Z15-SMA) for 7 days, and the aflatoxin contamination prevention effect was examined. The results showed that with increasing BS-Z15-SMA concentration, the aflatoxin contamination prevention effect was significantly enhanced. The above toxicity phenomena became more significant with extended BS-Z15-SMA treatment time. Scanning electron microscopy showed that 4 μg/mL Myco treatment resulted in a dented *A. flavus* surface and breakage of both the conidial stem and the mycelium. Transcriptome results showed that Myco significantly affected gene expression in *A. flavus* spores. The downregulated genes were significantly enriched in cell wall synthesis, transcription and translation, transmembrane transport pathways, and pathways related to key enzymes for aflatoxin synthesis. These results suggest that Myco could be used as a new bioactive material to prevent aflatoxin synthesis and contamination.

## 1. Introduction

*Aspergillus flavus* is a common saprophytic fungus. About 60% of all *A. flavus* strains produce aflatoxins [1] during their growth and development. They usually infect oil crops such as peanuts, corn, and rice, where they cause molds that ultimately result in aflatoxin contamination. Peanuts are the most susceptible oil crop to *A. flavus* infection [2]. Corn, which is also susceptible to *A. flavus* infection [3], is one of the most important cash crops and has the largest planting area in the world. Even after deep processing, the aflatoxins contained in contaminated corn will remain in related products, thus harming human and animal health [4,5]. Aflatoxins have received wide attention because of their high toxicity and carcinogenicity. Long-term aflatoxin exposure can lead to immunosuppression, poor nutritional metabolism, infertility, congenital malformations, endocrine disorders, and severe hepatocellular carcinogenesis in both humans and livestock [6]. Aflatoxin pollution is a serious threat to economic development and public health security, making it necessary to find effective prevention and control measures. With the rapid development of modern biotechnology, safer, more efficient, and environmentally friendlier biological control methods have gradually been adopted for the prevention and control of mold contamination. The most promising approach is the use of microorganisms. Microorganisms that have been found to inhibit *A. flavus* mainly include Bacillus, lactic acid bacteria, yeast, Streptomyces, Trichoderma, Pseudomonas, and certain marine microorganisms; these have been found to inhibit the growth of *A. flavus* by producing active substances such as peptides, bacteriocins, volatile organic compounds, organic acids, antibiotics, and enzymes [7]. Screening for strains with antagonistic effects against *A. flavus* is one of the research hotspots in the field of aflatoxin contamination prevention.

*Bacillus subtilis* is a Gram-positive bacterium that can form endogenous-resistant spores and features a fast growth rate, low nutrient requirements, and suitable growth conditions. During its growth, *B. subtilis* secretes large amounts of secondary metabolites with broad-spectrum antibacterial properties, such as subtilisin and polymyxin [8,9], which is why it has attracted considerable attention [10]. Li et al. [11] found that iturin A can damage the cell membrane of fungi. The resulting release of nucleic acid proteins has been found to inhibit the growth of *Fusarium fujikuroi* and *Botrytis cinerea*. Moyne et al. [12] isolated and purified bacillomycin D—a lipopeptide compound with an inhibitory effect on *A. flavus*—from the fermentation broth of *B. subtilis* AU195. Lulu found that metabolites of Bacillus could inhibit the growth of *A. flavus* on the grain surface [13]. Shifa et al. found that the *B. subtilis* G1 strain had an inhibitory effect on *A. flavus* in peanuts and soil and significantly inhibited *A. flavus* in soil, as well as *A. flavus* infection and aflatoxin B1 content in seeds, thus increasing pod yield [14]. The above studies indicate that the potential of *B. subtilis* as an important strain resource for preventing *A. flavus* contamination is promising.

When isolated in the early stage, the secondary metabolites of *B. subtilis* BS-Z15 have broad-spectrum-inhibiting fungal activity and can inhibit the growth of *A. flavus*. However, whether these secondary metabolites can be adopted for the prevention and control of *A. flavus* contamination in crops needs further scientific exploration. In this paper, the role of *B. subtilis* BS-Z15 secondary metabolites (BS-Z15-SMA) crude extract in the prevention of *A. flavus* contamination was studied. Further, the effects of the lipopeptide metabolite Mycosubtilin (Myco) obtained from the purification of BS-Z15-SMA [15] on the morphology and transcription level of *A. flavus* cells were also examined. The goal of this study was to clarify the mechanism underlying the growth inhibition of *A. flavus* by BS-Z15-SMA and to provide a theoretical basis for its application in the prevention of *A. flavus* contamination in oil crops.

## 2. Results

### 2.1. Effect of B. subtilis BS-Z15-SMA on the Prevention of A. flavus Contamination in Oil Crops

#### 2.1.1. Effects of *B. subtilis* BS-Z15-SMA on the Growth of *A. flavus* on the Corn Surface

**Figure 1:**
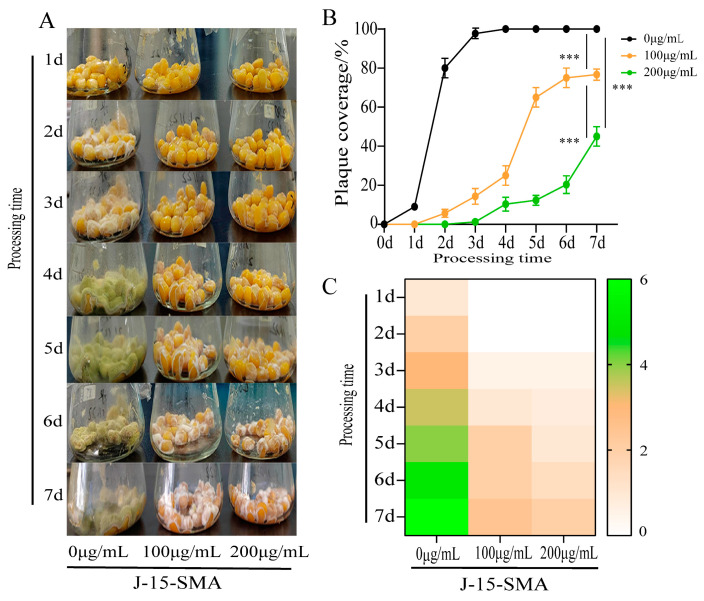
Effects of different concentrations of BS-Z15 secondary metabolites (BS-Z15-SMA) on preventing the growth of *Aspergillus flavus* on the corn surface. Note: Panel (**A**) is the result of different concentrations of BS-Z15-SMA to prevent the contamination of *A. flavus* on corn surface for 7 days; the more yellow/green these kernels look, the more they are contaminated; Panel (**B**) shows plaque coverage over time; Panel (**C**) shows a heat map of the effect of different concentrations of BS-Z15-SMA on preventing the growth and color change in *A. flavus* on the corn surface (‘***’ on the bar chart means *p* < 0.001).

In this study, after soaking corn with 100 μg/mL and 200 μg/mL BS-Z15-SMA, the growth of A. flavus on the surface of corn kernels was inhibited. An observation of colony color showed that in the control group, after inoculation with *A. flavus*, colonies began to grow within 1 d. The colonies gradually matured from white to yellow-green in color in 2–3 d, with basically no change after maturity. In the BS-Z15-SMA treatment group, although *A. flatus* showed growth, its growth stage remained in the white mycelium stage and did not enter the yellow-green mycelium maturity stage (Figure 1A,C). Compared with the control group, the growth of *A. flavus* in each of the treatment groups was significantly inhibited by BS-Z15-SMA, where the higher the BS-Z15-SMA concentration, the stronger the growth inhibition of the mycelium (Figure 1B). The above results showed that *B. subtilis* BS-Z15-SMA exerted a considerable inhibitory effect on the growth of *A. flavus* on the surface of corn.

#### 2.1.2. Effects of *B. subtilis* BS-Z15-SMA on the Growth of *A. flavus* on the Peanut Surface

**Figure 2:**
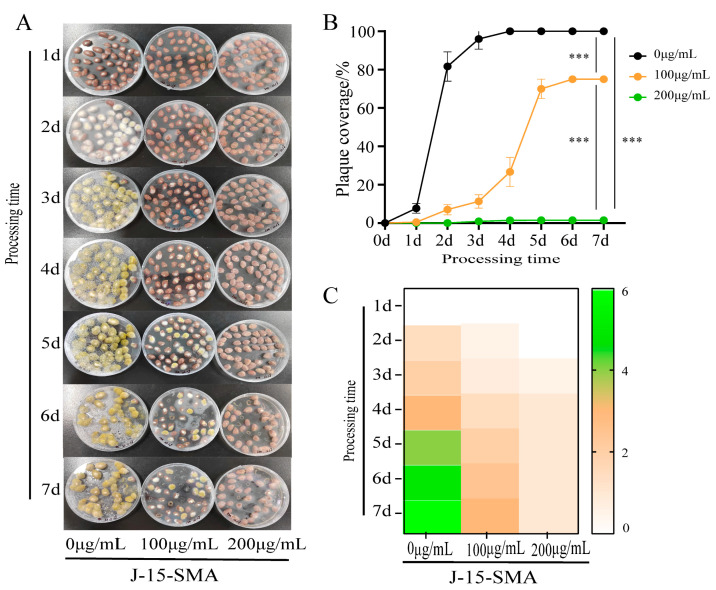
Effects of different concentrations of BS-Z15-SMA on the growth prevention of *A. flavus* on the peanut surface. Note: Panel (**A**) shows the inhibition results of different concentrations of BS-Z15-SMA on the contamination of *A. flavus* on the peanut surface for 7 days; Panel (**B**) shows the inhibition results of different concentrations of BS-Z15-SMA on the growth of *A. flavus* on the peanut surface over time; Panel (**C**) shows a heat map of different concentrations of BS-Z15-SMA preventing the color change in *A. flavus* growth on the peanut surface (‘***’ on the bar chart means *p* < 0.001).

When peanuts of the control group were inoculated with *A. flavus*, at 3 d, their surface was completely covered by *A. flatus*, and the colony showed yellow-green color at maturity. On the surface of peanuts treated with 100 μg/mL of BS-Z15-SMA, only a few *A. flavus* spores had germinated at 2 d. At 3 d, a few white colonies appeared on the surface, and significant colony expansion was observed at 4 d. At this time, the mycelium color changed from white to green, but the total area of colonies was significantly lower compared with the control group. The area of *A. flavus* plaque on the surface of peanuts basically did not change much at 5 d and 7 d, and the area of plaque did not expand or change color from white to green during this period (Figure 2A,C). Compared with the control group, peanuts treated with 200 μg/mL of BS-Z15-SMA only showed a small amount of *A. flavus* growth on the surface from 1 d to 7 d. The area of *A. flavus* on the peanut surface did not increase significantly over time. These results showed that with increasing BS-Z15-SMA concentration, the significant growth inhibition effect on *A. flavus* was stronger (Figure 2B). Although the growth was lower, the mold could grow at the maximum concentration.

### 2.2. Effects of Myco on the Morphology of A. flavus Spores and Mycelia

**Figure 3:**
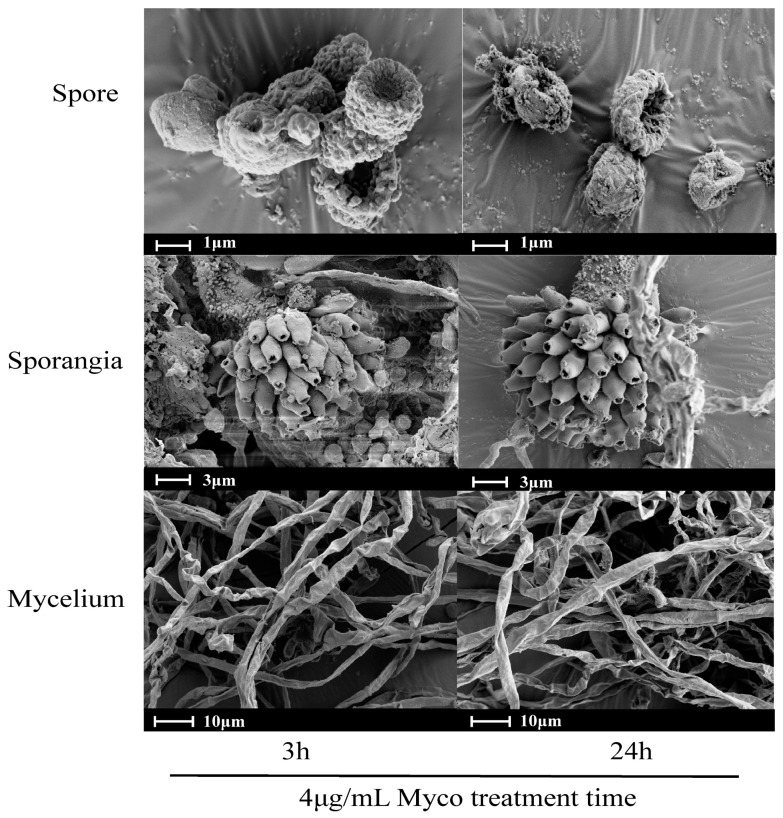
Effects of Myco treatment on the morphology of spores and mycelia of *A. flavus*. Note: Images of normal spores and sporangia of the control group are obtained from the Internet (https://health.sina.cn/iw/aiwenArticle/5902b9d50cf2a0e37782f722?contentType=2, accessed on 25 May 2022), and the image of normal mycelium is obtained from Fangce [16].

The morphology of spores and mycelia of *A. flavus* treated with 4 μg/mL Myco for 3 h was observed by scanning electron microscopy. Compared with the control group, the cell wall surface of several *A. flavus* spores were concave and “bowl-like”, the surface granular protrusion was worn, the top conidial pedicle was broken, and the mycelia shape was no longer full but wrinkled, collapsed, and twisted. Multiple mycelia had broken, and the contents had flowed out (Figure 3). With extended Myco treatment time, the toxic effects became more apparent. This result indicates that Myco may cause a change in the membrane permeability of *A. flavus* spores, the disruption of the sporangium structure, a change in mycelium morphology, a decline of sporulation ability, and finally, death.

### 2.3. Transcriptome-Based Analysis of the Mechanism Underlying Myco-Induced Growth Retardation of A. flavus

#### 2.3.1. Quality Control and Correlation Analysis of Transcriptome Data

Q30 is a measure of NGS data quality, which indicates the percentage of sequence potential window data quality score during conventional chemical amplification (cDNA), thus representing a strong statistical signal. Generally speaking, the higher the score of Q30, the higher the quality of the result detection and the better the technology. The Q30 value of all samples was higher than 93.71%, the GC content distribution was qualified (Table S1), the sample sequence data quality was high, and the intra-group consistency and inter-group difference were suitable. These results indicate that these data can be used for bioinformatics analysis. The gene sequence information is shown in Table S1 in the Supplementary Materials, and a correlation analysis among the treatment groups is shown in Figure S1 in the Supplementary Materials.

#### 2.3.2. Statistics of Differentially Expressed Genes

**Figure 4:**
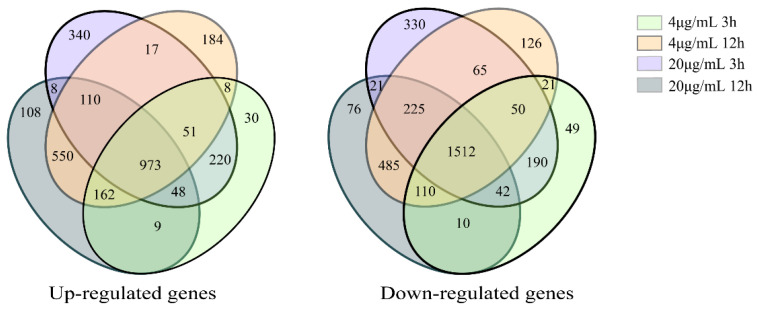
Venn diagram of differentially expressed genes of *A. flavus* treated with different Myco.

The expression of different genes was calculated using the threshold of significance level (*p*-value < 0.05) and the absolute value of log2Ration ≥ 2 to determine the number of differentially expressed genes in different groups. A total of 2485 differentially expressed genes were identified across the different groups treated with 4 μg/mL and 20 μg/mL Myco. There were 973 upregulated genes and 1512 downregulated genes (Figure 4).

#### 2.3.3. GO Classification and KEGG Enrichment Analysis of Differential Gene Expression after Myco Treatment of *A. flavus*

**Figure 5:**
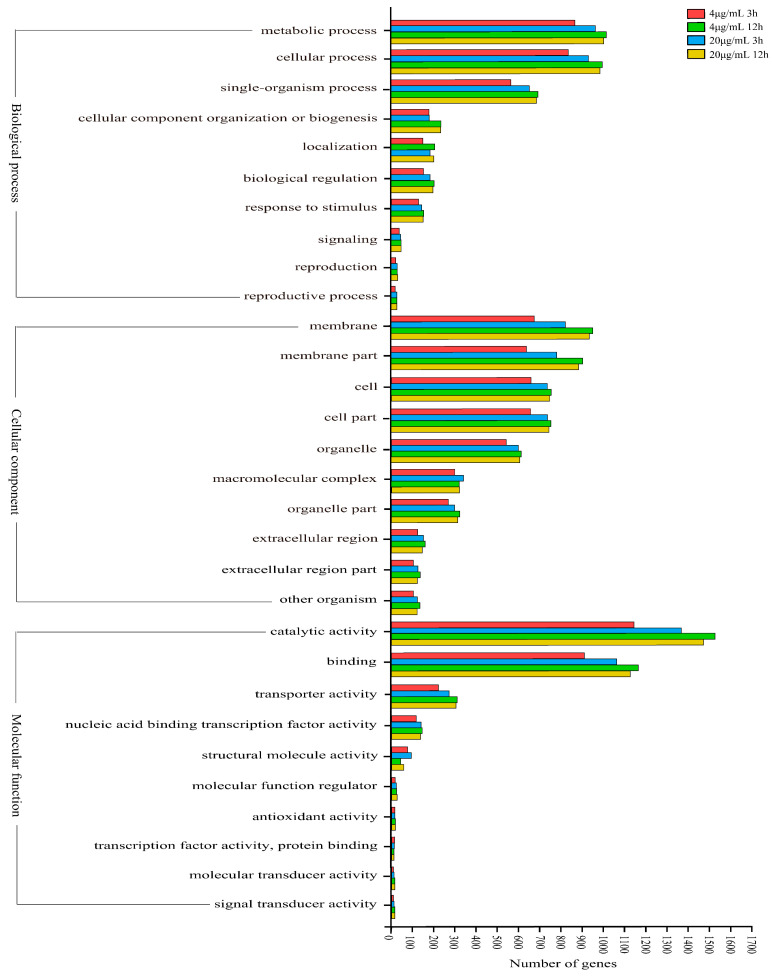
GO classification map of differentially expressed genes in different groups of *A. flavus* treated with Myco.

GO classification analysis showed that the number of differentially expressed genes increased with increasing Myco concentration under the same treatment duration. Under the same Myco concentration, the number of differentially expressed genes increased with extended treatment time. In biochemical processes, differentially expressed genes were mainly classified into metabolic processes, cellular processes, monobiotic processes, cell substance organization, and biogenesis and motility. In cell substances, differentially expressed genes were mainly classified into membrane and membrane substances and extracellular and macromolecular complexes. In molecular function, differentially expressed genes were mainly classified into catalytic activity, binding activity, transport activity, nucleic acid-binding transcription factor activity, and structural molecular activity (Figure 5). Therefore, according to the results of GO classification, Myco treatment can affect substance synthesis, cell membrane function, transcription, and translation, thus affecting the growth of *A. flavus*.

**Figure 6:**
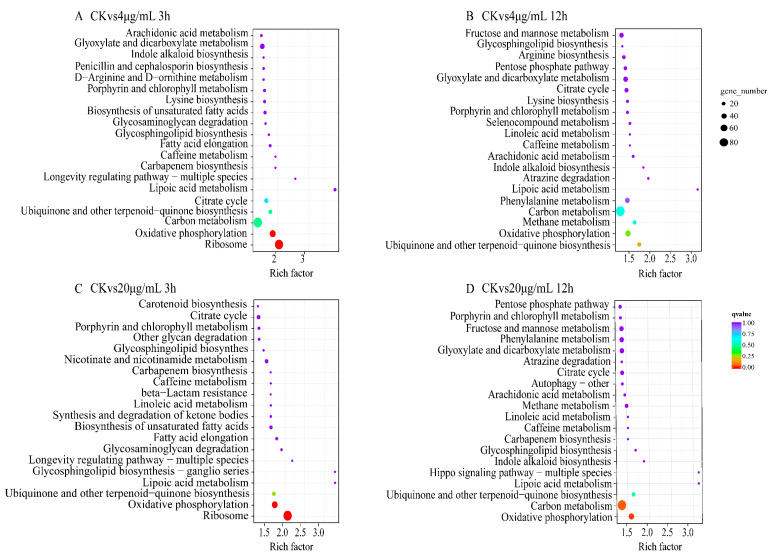
KEGG enrichment results of differentially expressed genes in different groups of *A. flavus* treated with Myco. (**A**) shows the KEGG enrichment map of the differentially expressed genes in the control group in the 4 μg/mL and 3 h treatment group; (**B**) shows the KEGG enrichment map of the differentially expressed genes in the control group and the 4 μg/mL and 12 h treatment group; (**C**) shows the KEGG enrichment map of differentially expressed genes in the control group treated with 20 μg/mL for 3 h; (**D**) shows the KEGG enrichment map of differentially expressed genes in the control group and the 20 μg/mL treatment group for 12 h.

KEGG pathway enrichment analysis of differentially expressed genes (Figure 6) showed that different Myco treatment groups were significantly enriched in oxidative phosphorylation, ribosome, carbon metabolism, biosynthesis of ubiquitin-ketone and other terpenoid quinones, lipoic acid metabolism, and tricarboxylic acid cycle metabolic pathways. These results indicated that Myco treatment affected the synthesis, energy metabolism, material circulation, and antioxidant function of *A. flavus* cells; moreover, their normal physiological activities were inhibited, cell resistance was weakened, and the growth and development of *A. flavus* cells were adversely affected.

#### 2.3.4. Myco Downregulates the Expression of Transporter Genes on the Membrane of *A. flavus*

**Figure 7:**
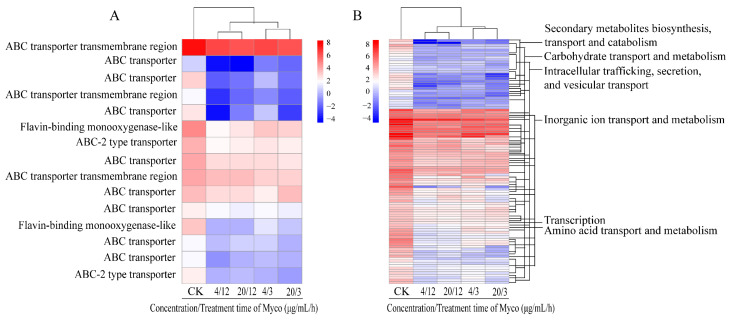
Clustering heat map of the expression levels of *A. flavus* cell membrane transporter-related genes regulated by Myco. (**A**) shows an ABC transporter protein; (**B**) shows a major facilitator superfamily (MFS) protein.

The scanning electron microscope images showed that Myco treatment negatively affected mycelium formation, and mycelia were wrinkled, collapsed, and twisted; moreover, multiple mycelia had broken, and the contents had flowed out, indicating that Myco had a strong effect on membrane permeability of *A. flavus*. Therefore, the expression of transporter-related genes on the cell membrane was further analyzed after Myco treatment. The results showed that the transcription levels of 102 genes of the major facilitator superfamily (MFS) protein and 15 genes of the ABC transporter in *A. flatus* were downregulated after Myco treatment (Figure 7). The transcription levels of ABC-2 transporter, ABC transporter, flavin monooxygenase, and ABC transmembrane transporter in the ABC transporter were mainly downregulated after Myco treatment. The transcription levels of amino acid transport and metabolism in MFS proteins, transcription, biosynthesis of secondary metabolites, transport and catabolism, intracellular transport, secretion and vesicular transport, transport and metabolism of carbohydrates, transport of inorganic ions, and transcription of metabolic protein-related genes were downregulated. The above results indicate that treatment with Myco can affect the expression of transporters on the *A. flavus* membrane. These expression changes result in changes in the number of transporters, thus changing the channels on the membrane. Consequently, the information transmission, material exchange, and energy metabolism of cells are changed, ultimately affecting the growth and development of *A. flavus*.

#### 2.3.5. Myco Downregulates the Expression of Cell Wall-Related Genes in *A. flavus*

**Figure 8:**
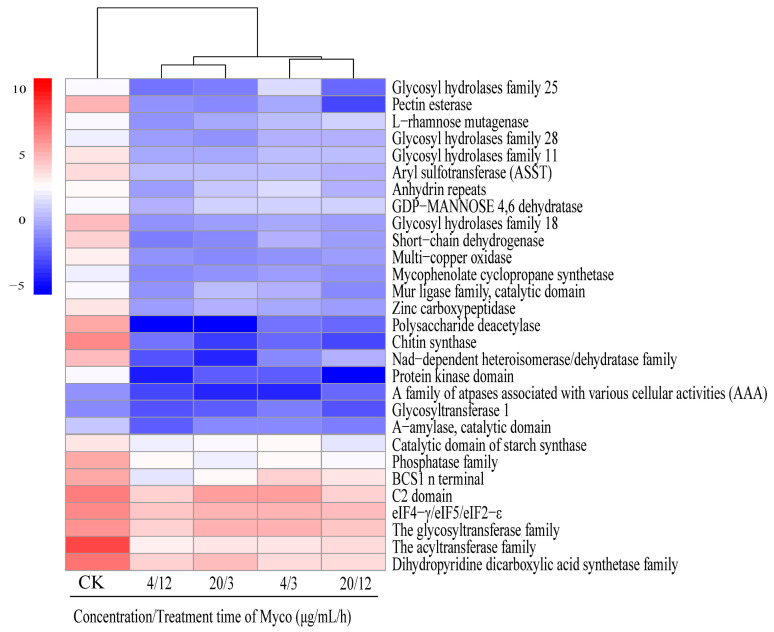
Clustering heat map of gene expression levels related to the regulation of cell wall synthesis in *A. flavus* by Myco.

Scanning electron microscopy showed that Myco exposure led to a concave and “bowl-like” phenotype of the surface of *A. flavus* cell walls. The surface granular protrusion was worn, the top conidial stem was broken, and spore production decreased, indicating that Myco substantially affected the morphology and function of the *A. flavus* cell wall. Analysis of the differentially expressed genes showed downregulated expression of 29 genes associated with the *A. flatus* cell wall (Figure 8). These genes include chitinase synthase, ATPase family (AAA) associated with multiple cell activities, acyltransferase, NAD-dependent differential isomerase/dehydrase family, short-chain dehydrogenase, GDP mannose dehydrogenase family, NAD-binding domain, and other genes.

#### 2.3.6. Myco Downregulates the Expression of Transcription Translation-Related Genes in *A. flavus*

**Figure 9:**
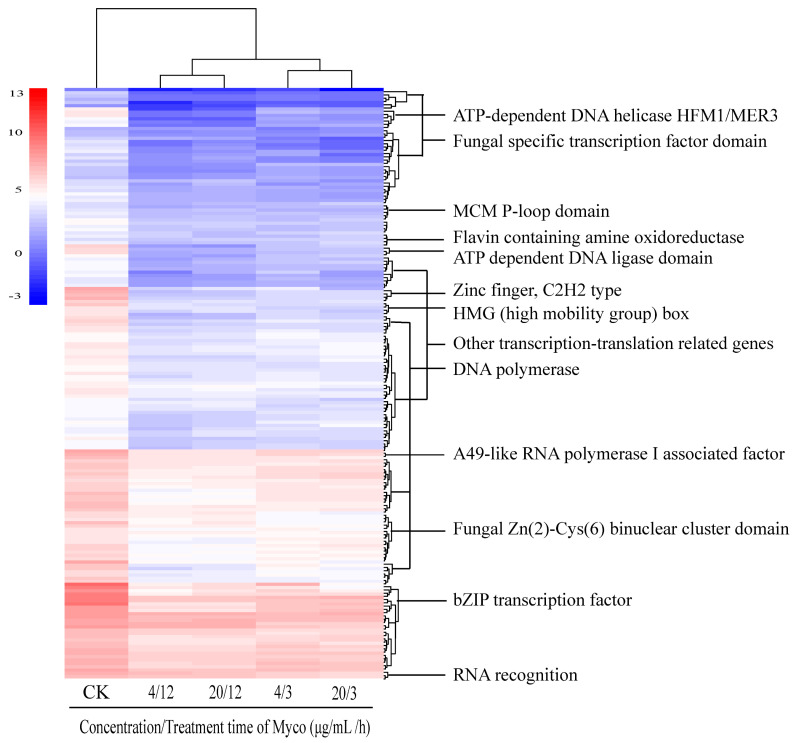
Clustering heat map of the expression levels of genes related to transcription in *A. flavus* regulated by Myco.

Following Myco treatment, analysis of differentially expressed gene sets showed that 209 genes related to transcription translation were downregulated (Figure 9). These genes include fungus-specific transcription factor domains, anti-codon binding domains, bZIP transcription factors, and RNA recognition groups. These gene expression changes indicated that Myco affects the transcription and translation process in *A. flavus* and damages cells by blocking the transcription and translation of DNA and RNA. The downregulated expression of these genes affected the synthesis of substances, resulting in an insufficiency of substances cells need and inhibition of overall cell activity. Among the identified differentially expressed genes, no genes related to apoptosis and necrosis were detected, and only a few genes were related to early apoptosis. Therefore, it can be speculated that the mechanism of Myco inhibition of *A. flavus* is not the triggering of apoptosis or necrosis but the damage of cells by interfering with the synthesis of substances, thereby inhibiting the growth of cells.

#### 2.3.7. Myco Downregulates Genes Related to Aflatoxin Synthesis

**Figure 10:**
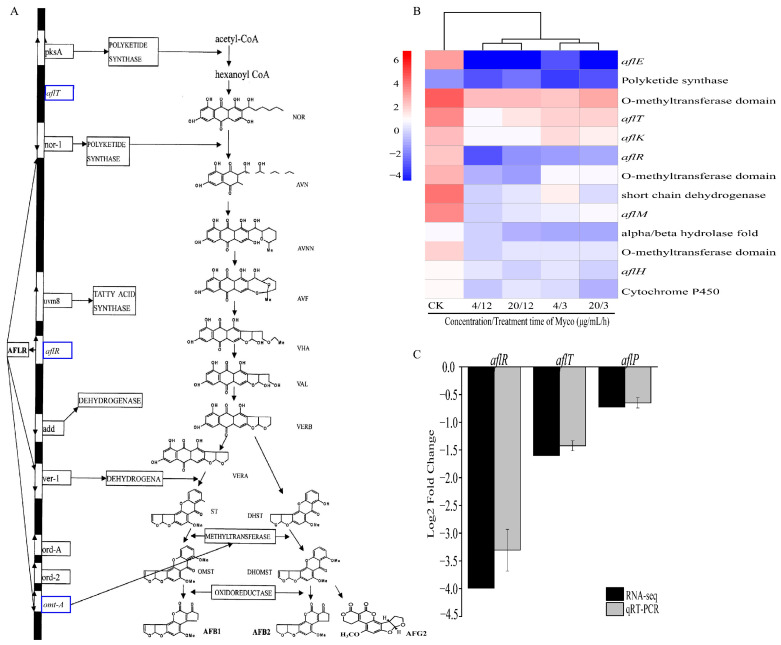
Effects of different Myco treatments on genes related to aflatoxin synthesis. (**A**) shows the effects of different Myco treatments on aflatoxin synthesis genes (the image is adapted from Xu and Luo [17]). The blue box represents the downregulation of related genes; (**B**) clustering heat map of gene expression related to toxin synthesis; (**C**) shows the RT-qPCR validation of the result of Myco treatment in *A. flavus*.

After Myco treatment of *A. flavus*, 13 genes in the aflatoxin synthesis pathway were downregulated, including *aflR*, which is the key regulatory gene of aflatoxin synthesis, and also *aflE*, *aflH*, *aflK*, *aflT*, and *aflM*. In addition, the gene expressions of short-chain dehydrogenase, cytochrome p450 enzyme, O-methyltransferase, and polyketosynthetase related to aflatoxin synthesis were also significantly downregulated (Figure 10B). These genes regulate several important steps in the aflatoxin synthesis pathway (Figure 10A), and their downregulation will affect aflatoxin production and inhibit the activity of *A. flavus* cells.

The *aflR*, *aflP*, and *aflT* genes necessary for cell growth and aflatoxin production in *A. flatus* were confirmed by RT-qPCR, and the results showed that all genes were downregulated after Myco treatment (Figure 10C). This result was consistent with the results obtained by transcriptome screening. The content of aflatoxin G2 in peanuts was determined using the high-performance liquid chromatography post-column derivatization method. The results are shown in Table 1. BS-Z15-SMA can effectively inhibit the synthesis of aflatoxin by aflatoxin on the surface of peanuts, and the AFG2 content in the two treatment groups decreased from 3.4 pbb in the control group to 0. The results indicate that BS-Z15-SMA can inhibit the synthesis and secretion of aflatoxin.

## 3. Discussion

### 3.1. BS-Z15-SMA Can Prevent A. flavus Contamination in Oil Crops

*A. flavus*-contaminated food seriously harms the human body. This study showed that *B. subtilis* BS-Z15-SMA can prevent the contamination of *A. flavus* in corn and peanuts by significantly inhibiting the growth of *A. flavus* in these oil crops. According to Hongbin et al., the color of *A. flavus* colonies changes from white to yellow and yellow-green with their growth, and the color turns brown after spores have matured [18]. *B. subtilis* J15-SMA arrests the growth of *A. flavus* at the white mycelium stage, effectively preventing cells from growing to maturation, thus inhibiting the growth and germination of *A. flavus* spores on corn and peanuts. The inhibitory effect of *B. subtilis* BS-Z15-SMA was stronger within a concentration range of 0–200 μg/mL, and this inhibitory effect was positively correlated with the concentration of *B. subtilis* BS-Z15-SMA. Kong et al. [19] applied the liquid culture product of a screened strain of *Bacillus gigantus* to peanuts, which reduced the incidence of *A. flavus*; within the set range of their experiment, with increasing bacterial concentration and extended culture time, the inhibiting fungal effect was consistently enhanced. This finding indicates that the metabolites of Bacillus are the main substances inhibiting the growth of *A. flavus*. However, whether the prevention effect of aflatoxin contamination in corn and peanuts increases with further increasing concentrations of *B. subtilis* BS-Z15-SMA still needs further experimental verification.

Transcriptome analysis showed that several genes in the aflatoxin synthesis pathway were downregulated after treatment with Myco: the inhibiting fungal active substance of *B. subtilis* BS-Z15, including *aflR*, the key regulatory gene of aflatoxin synthesis, and the downstream regulatory genes *aflE*, *aflH, aflK*, *aflT*, and *aflM*. The *aflR* gene is one of the most important regulatory and initiatory genes in aflatoxin biosynthesis and encodes the 47 kDa protein AFLR. During aflatoxin synthesis, AFLR activates the transcription of the downstream genes *nor-1*, *wer-1*, *omt-A*, and other structural genes in the aflatoxin synthesis pathway, i.e., the transcriptional activity of downstream genes depends on the transcription of *aflR* genes [17]. AFLR regulates aflatoxin production at the transcriptional level and is an important transcriptional activator in aflatoxin biosynthesis. Downregulation of its expression directly affects the syntheses of aflatoxin B1 and aflatoxin B2, and it has been found that *Bacillus amyloliformis* WF2020 can inhibit fungal growth and reduce the production of aflatoxin B1. The expressions of aflatoxin pathway genes and two transcription factors (*aflR* and *aflS*) were downregulated consistently [20]. The omtA (*aflP*) gene encodes methyltransferase, which is involved in the methylation of many precursor substances in the aflatoxin synthesis pathway, and also plays an important role in aflatoxin synthesis. Liang [21] found that eugenol can significantly downregulate the expression of the *omtA* gene, resulting in a reduction in the corresponding translation product transmethoxylase, thus blocking the synthesis path of catalytic precursors and inhibiting the production of aflatoxins. Liang also showed that Myco downregulates the expression of key genes in the *aflR* synthesis pathway and key enzymes in subsequent synthesis pathways (such as polyketosynthetase and methyltransferase), thus jointly inhibiting aflatoxin production. Prior studies have found that aflatoxins, the secondary metabolites of *A. flavus*, not only affect the surrounding environment through their high toxicity but also improve the survival rate of spores. Aflatoxin secretion is closely timed with spore germination, and reduced aflatoxin secretion will lead to slower spore formation. When aflatoxin secretion is completely inhibited, spore production is basically zero [22].

Myco can not only inhibit the growth of *A. flavus* but can also act on the synthesis site of aflatoxin. It can also inhibit the formation of spores while attenuating the toxin, which has great potential to be developed into an efficient, environmentally friendly, and economic anti-mold preparation. In a follow-up experiment, the authors will examine whether the metabolites of *B. subtilis* BS-Z15 could inhibit the accumulation of aflatoxin in grains.

### 3.2. Myco Has Toxic Effects on A. flavus Cells

The surface of normal *A. flavus* cells shows clear protrusion, appropriate spore morphology, broom-like sporangium, the formation of multiple immature spores at the top conidial stem, thriving mycelium, full shape, and smooth surface. However, after treatment of *A. flavus* with Myco, the cell wall surface appears concave and “bowl-like”, and the surface granular protrusion appears worn. These results showed that Myco had toxic effects on *A. flavus* cells, and the mycelium showed a crumpled, collapsed, and twisted morphology. The mycelium was broken in many places, and its contents flowed out. Lulu’s research showed that volatile substances of Bacillus could change the mycelium morphology of *A. flavus*, causing mycelium adhesion and surface collapse [13]. Changes in cell wall morphology will inevitably lead to changes in cell membrane permeability; in microbial life activities, the cell wall and cell membrane undertake many important physiological activities. These activities are of great importance for material exchange, information exchange, and maintenance of cell morphology inside and outside of cells. Changes in cell permeability are not conducive to the normal life of microorganisms [23]. Proteases produced by *Bacillus amylus* and *B. subtilis* have been shown to affect fungal growth and aflatoxin production by changing the permeability of *A. flavus* cells, penetrating into the cytoplasm, and destroying organelles [24]. Another study found an antagonistic strain of Bacillus with clear anti-*A. flavus* activity; the lipopeptides (LPs) produced by this strain could inhibit spore germination and even cause abnormal hyphal expansion and cell rupture [25]. The inhibitory effect of Myco on *A. flavus* is the same as that found in other studies. Therefore, it can be speculated that Myco may mainly change the cell wall and cell membrane morphology of *A. flavus*, downregulate the expression of genes related to the cell wall and cell membrane, and thus inhibit the growth and reproduction activities of *A. flavus*.

Transcriptome analysis found that after Myco treatment, the expression levels of genes related to short-chain dehydrogenase, chitinase synthase, ATPase family (AAA), acyltransferase, NAD-dependent heterotrophy isomerase/dehydrase family, GDP mannose dehydrogenase family, and NAD-binding domain related to cell wall synthesis were significantly downregulated. This downregulation can affect *A. flavus* cell wall synthesis [26]. The fungal cell wall is composed of chitin and polysaccharides, and with increasing Myco concentration and extended treatment time, the gene for chitin synthesis was significantly downregulated. This result indicates that Myco can interfere with the cell wall synthesis of *A. flavus*, causing the cell wall to deform or disrupt, thus significantly decreasing the resistance to external stimulation and ultimately leading to the reduction of cell activity and even cell death [27,28,29]. The energy hydrolysis of the AAA protein originates from the hydrolysis of ATP in its AAA domain, which plays an important role in many cellular processes, including protein development and degradation, membrane fusion, nucleosome remodeling, and microtubule disassembly; thus, it participates in the regulation of various physiological and pathological processes [30]. As an important part of the cytoskeleton, the microtubule skeleton plays a key role in many physiological activities, such as cell division and cell growth [31]. Following Myco treatment, in *A. flavus*, AAA genes related to various cell activities were downregulated, which affected various cell activities, such as inhibiting cell wall formation and spore growth. The fungal cell wall also maintains normal cell metabolism, conducts ion exchange with the extracellular space, and regulates osmotic pressure [32]. The results of scanning electron microscopy of *A. flavus* cells treated with Myco are consistent. In conclusion, Myco has toxic effects on *A. flavus* cells. By affecting the activity of the *A. flavus* cell wall, it causes cell dysfunction, changes in cell osmotic pressure, and loss of the ability to maintain homeostasis. Finally, the growth of *A. flavus* was inhibited, and new spores could not be produced.

At present, it is known that lipopeptin antibiotics produced by *B. amylus* B10-6-1 and antagonistic proteins in *Bacillus aerobacillus* Y-17-3 have antibacterial effects by inhibiting spore germination and mycelium growth in *A. flavus* [33,34]. The results of this study also showed that Myco inhibited the growth and production and changed the morphology of *A. flavus* cells. The transcriptomic results of the *A. flavus* morphology and the downregulation of genes related to toxigenesis further demonstrated the inhibitory effect of Myco on *A. flavus*. Future studies will focus on the site of action of Myco in *A. flavus* cells.

### 3.3. Myco Affects the Material Metabolism and Energy Metabolism of A. flavus

Wei [27] found that cuminaldehyde can inhibit the growth of *A. flavus*, affect its biosynthesis and metabolism, regulate its growth and development, and downregulate the biosynthesis of conidium (the principal substances of the cell membrane) as well as its related transport proteins and cell cyclin. Transporter-related genes on the membrane of *A. flavus* were also significantly downregulated after Myco treatment. Many membrane transporters are involved in regulating the flow of molecules in and out of fungal cell membranes, including the ATP binding cassette (ABC) superfamily and major facilitator superfamily. MFS is a typical transporter superfamily. The diversity of MFS protein transport substrates ensures that they play an important role in the processes of cellular material exchange and energy metabolism. They can transport many small molecules, such as monosaccharides, polysaccharides, amino acids, peptides, vitamins, enzyme cofactors, drug molecules, chromophores, and bases [35,36,37,38]. ABC transporters are a type of membrane integrin that widely exist in the biological world; they can catalyze the hydrolysis of ATP and use the energy thus generated to promote the transmembrane transport of substrates [39]. ABC superfamily proteins are typically multisubstances transporters that are capable of transporting small and large molecules across membranes under ATP hydrolysis. They transport a wide range of compounds: polysaccharides, drugs, sugars, heavy metals, oligopeptides, amino acids, and inorganic ions [40]. The flavin monooxygenase in the ABC protein is widely involved in biological reaction processes and plays a key role in compound metabolism [41]. Most flavin monooxygenases catalyze the formation of covalent bonds between flavin C4a and molecular oxygen to produce flavin peroxide intermediates, which can further oxidize the target substrate under the action of flavin monooxygenase [42]. The transcription levels of the ABC-2 transporter, ABC transporter, flavin monooxygenase, and ABC transmembrane transporter in the ABC transporters of *A. flavus* were mainly downregulated after Myco treatment. Their downregulation inhibited the transport of small and large molecules, energy metabolism, compound metabolism transformation, and various biological reactions. Transport and metabolism of amino acids, transcription, biosynthesis of secondary metabolites, transport and catabolism, intracellular transport, secretion and vesicular transport, transport and metabolism of carbohydrates, transport of inorganic ions, and transcription levels of metabolic protein-related genes in MFS proteins were all downregulated. These results indicated that Myco inhibited normal physiological metabolic activities, such as the synthesis and processing of proteins, energy metabolism, information transfer, and substance synthesis. Further, the exchange of substances inside and outside of *A. flavus* cells, information transfer between cells, and energy metabolism were inhibited, the activity of *A. flavus* cells and aflatoxin synthesis were reduced, and the transformation of substances was hindered. Eventually, these effects can lead to cell death and inhibit the growth of *A. flavus*.

The secretion of aflatoxins is a response to external stimuli. If excessive reactive oxygen species and a large number of carbon cycle intermediates accumulate in cells, *A. flavus* can metabolize aflatoxins and expel the excess out of cells [43]. After Myco treatment, the expression of several regulatory genes in the aflatoxin synthesis pathway was downregulated, which may cause the accumulation of a large amount of aflatoxin-synthesized substances in cells. Accumulation of these substances is not conducive to the normal metabolic activities of *A. flavus* cells, and excessive accumulation may even cause cell death [44,45,46]. The downregulation of fungus-specific transcription factor domains, anti-codon binding domains, bZIP transcription factors, and RNA recognition groups related to transcription translation suggests that Myco can directly affect the transcription translation of *A. flavus* genes. By inhibiting transcription translation, Myco can slow down the processes of cell division and substance synthesis in *A. flavus*. Consequently, the basic physiological activities, cell growth, and cell division of *A. flavus* cells cannot proceed normally, thus inhibiting the growth of *A. flavus*.

Wu et al. found that after treatment of *A. flavus* with Bacillus B2, genes of the energy metabolism pathway, transcription, folding, and transport pathway, biosynthesis, and catabolic pathway of *A. flavus* were downregulated; they suggested that the active substances secreted by Bacillus B2 caused protein damage and membrane structure destruction in *A. flavus* and that *A. flavus* synthesized proteins through other ways to maintain its basic growth capability; downregulated genes may affect ribosome assembly and protein synthesis, resulting in inhibited growth and reduced aflatoxin biosynthesis [47]. This finding is consistent with the inhibitory effect of Myco on *A. flavus*. Myco also affects the material metabolism and energy metabolism of *A. flavus* from various aspects, ultimately inhibiting the growth and reproduction of *A. flavus*. Further studies can explore the effect of Myco on preventing *A. flavus* pollution in cereals and its development into a stable and efficient anti-mildew preparation.

## 4. Conclusions

*B. subtilis* BS-Z15-SMA could prevent *A. flavus* contamination of corn and peanuts, and the inhibitory effect was enhanced with increasing BS-Z15-SMA concentration. Treatment of *A. flavus* with 4 μg/mL Myco resulted in spore surface depression and shrinkage, top conidial stem fracture, decreased spore production capacity, as well as shrinkage, collapse, and distortion of the mycelium. These toxic phenomena were stronger with extended treatment time. The Myco-induced inhibition of *A. flavus* was not caused by a single function but by the regulation of the expression of transporters, cell wall, transcription translation, and toxin synthesis-related genes in the membrane of *A. flavus*. In summary, the development of Myco as an anti-aflatoxin preparation has a suitable basis. Myco has great potential to be developed into an efficient, environmentally friendly, and economic antifungal preparation.

## 5. Materials and Methods

### 5.1. Experimental Materials

In this study, *B. subtilis* BS-Z15 was independently isolated and purified by our laboratory, enriched and cultured with beef extract peptone medium, and its metabolites BS-Z15-SMA and Myco at different stages were extracted and used for backup according to Lin Rongrong’s [15] method. *A. flavus* species BNCC336678 was purchased from Beijing Beina Chuanglian Biotechnology Research Institute (Beijing, China). The Potato Glucose broth (PDB), Potato Glucose Agar (PDA), and beef extract peptone used were purchased from Xinjiang Baoxin Yuanbai Biotechnology Co., Ltd. (No. B3-356, Dongyi Lane, North Station Second Road, Xinshi District, Urumqi, Xinjiang, China). Corn and peanuts are purchased in supermarkets (125 Xinyi Road, Xinshi District, Urumqi, Xinjiang, China).

### 5.2. Preparation of A. flavus Spore Suspension

After the *A. flavus* colony grew and matured, small pieces of mycelia were cut and inoculated into PDB liquid medium and cultured at 180 rpm/min at 28 °C for 3–4 days. After small globular mycelia appeared in the liquid medium, globular mycelia was filtered, and a single mycelium group was inoculated onto the surface of the PDA plate until the colony grew to yellowish green. The spores were washed by repeated blowing with 5 mL sterile water, and the mycelium was removed by filtering the blowing solution with sterile cotton cloth. The spore solution was rinsed with PBS at 19,347× *g* at 4 °C and centrifuged to make suspension cells. After full shock, the concentration of spore solution was calculated by calculating the concentration of the spore solution using a hemocytometer, and the concentration was diluted to (1 × 10^6^ CFU/mL).

### 5.3. Treatment of Corn and Peanut Samples by BS-Z15-SMA

A number of corn and peanut samples were randomly taken. Each 50 mL conical bottle was separately divided into 10 g edible corn, and each petri dish was placed with 30 g edible peanuts, washed with distilled water three times, removed, sterilized by high-pressure steam at 121 °C for 20 min, cooled to room temperature, and randomly divided.

Control group: Sterilized peanuts and corn were soaked in 500 μL sterilized water for 12 h and then inoculated with (1 × 10^6^ CFU/mL) *A. flavus* spore suspension. Each group of corn was inoculated with 50 μL. Inoculate each peanut surface with 0.5 μL.

Treatment group: The sterilized peanuts and corn were soaked with 500 μL BS-Z15-SMA solution with concentrations of 100 μg/mL and 200 μg/mL, respectively, for 12 h and then inoculated with the control group. After inoculation, it was cultured in an incubator at 28 °C, and the growth of *A. flavus* was recorded by taking photos every 24 h.

### 5.4. Effects of Myco on the Morphology of A. flavus Spores and Mycelia

Due to the difference in purification efficiency of BS-Z15-SMA, 20 μg Myco can be purified from 200 μg BS-Z15-SMA at this time. In the previous experiments to prevent *A. flavus* grain contamination, the semi-lethal concentration of Myco on the model fungus yeast (*S. cerevisiae* S288C) was 4 μg/mL [48]. Based on comprehensive consideration, 4 μg/mL and 20 μg/mL Myco were selected in this experiment to explore their effects on the morphology of *A. flavus* spores and mycelia.

1 mL suspension was taken, and then 1 mL 4 μg/mL Myco solution was added into the Ep tube, and each group was cultured for 0 h, 3 h, and 24 h, respectively. Myco solution was removed after 19,347× *g* centrifugation at 4 °C for 5 min and rinsed three times with PBS. A total of 1 mL 2.5% glutaraldehyde solution was added to each group, which was preserved and fixed at 4 °C for 24 h, rinsed three times with PBS, and then dehydrated once with 30%, 60% and 90% concentration ethanol solution in gradient. Then, the samples were dehydrated with 100% ethanol 3 times, each time for 30 min. The samples were replaced in tert-butanol solution for 30 min and then freeze-dried in a freeze-drying machine for 3 h to produce SEM samples. Before taking pictures on the scanning electron microscope, a small amount of sample was picked up with a toothpick and adhered to the conductive adhesive, and the floating dust was carefully blown away with the ear wash ball. Since the sample was not conductive, it was necessary to spray gold on the sample with a paste sputtering meter and observe and take pictures with the scanning electron microscope.

### 5.5. Treatment of Transcriptome Spore Samples

The *A. flavus* spore suspension was centrifuged at 4 °C for 19,347× *g* for 5 min, the supernatant was discarded, and the spore weight was weighed. For every 100 mg of spores, 1 mL of Myco solution with a concentration of 4 μg/mL and 20 μg/mL were added, respectively. *A. flavus* spores were cultured at 180 rpm/min at 28 °C for 3 h and 12 h, centrifuged at 19,347× *g* for 5 min to remove the culture medium, rinsed with 500 μL sterile water three times, centrifuged, frozen with liquid nitrogen and stored in a freezer at −80 °C.

### 5.6. RNA Extraction, Library Construction, and Sequencing Analysis

The total RNA of *A. flavus* was extracted by the Trizol method. Add 1mL Trizol into a 1.5 mL centrifuge tube without RNAase for use. Take the mycelium about 1 g in liquid nitrogen and quickly grind it to powder. Add the corresponding amount of sample to the centrifuge tube with Trizol. Shake the sample and the extraction solution and let it stand at room temperature for 5 min to completely decompose the nucleic acid protein complex. Add 200 μL of chloroform, quickly shake, and mix for 15 s. Let stand at room temperature for 3–5 min at 4 °C, 19,347× *g* for 15 min, carefully absorb about 400 μL supernatant into the new centrifuge tube. Add 0.5 mL isopropyl alcohol into the upper liquid, shake gently, and precipitate at room temperature for 10 min. Centrifuge at 19,347× *g* at 4 °C for 10 min, white precipitate appeared on the bottom of the tube. After removing the supernatant, add 75% ethanol prepared with DEPC water into the centrifugal tube, and use the pipette to suck and wash the precipitation. Centrifuge at 8598× *g* at 4 °C for 5 min, remove the supernatant, and dry on filter paper at room temperature for 15–20 min. A total of 45 μL RNase-free water-dissolved RNA was added to the tube and stored at −80 °C after complete dissolution. Obtain RNA samples with purity standards using a nucleic acid detector Nanodrop2000 (OD260/OD280 ≥ 1.8, OD260/OD230 ≥ 1.5), detect RNA Integrity Index (RIN) ≥ 8.0, and evaluate the quality and purity of RNA samples using RNA 6000 Nano LabChip reagent kit and Bioanalyzer 2100 (Agilent Technologies, Santa Clara, CA, USA). PCR was used to amplify and create the final cDNA library. The Illumina HiSeq 2000 platform was used for high-throughput fragment sequencing of the final cDNA library created, and the sequenced data were filtered to obtain clean data, and subsequent data analysis was conducted.

### 5.7. Quantitative and Differential Expression Analysis of Gene Expression Levels

After the Read counts of the genes/transcripts were completed, each gene reading was calculated using feature counts, and the pair-to-pair comparison combinations were analyzed using DESeq2R software (1.16.1) [49]. The *p*-value was calculated by the Benjamini and Hochberg method and adjusted. When *p* adjust < 0.05, significant enrichment was considered.

### 5.8. Bioinformatics Analysis of Differentially Expressed Genes

The sequenced genes and transcripts were compared and annotated with six public databases (NR [50], Swiss-Prot [51], COG [52], KEGG [53], GO [54], Pfam [55]) one by one, and the annotated information of all genes in each database was statistically analyzed after the details were obtained. The genes with significant differences were classified by GO and analyzed by KEGG enrichment. When *p*-value < 0.05 was adjusted, the genes were significantly dot after enriched.

### 5.9. Transcriptome RT-qPCR Validation

RT-qPCR was used to verify the high-throughput sequencing analysis results, refer to the specific primers designed for the functional gene sequence of *A. flavus* [16], and commission Bioengineering (Shanghai, China) Co., Ltd. to complete the synthesis, using *18S* gene as the internal reference gene. The RT-qPCR primer sequence, reaction system, and process are shown in Tables S2–S4 in the Supplementary Materials.

### 5.10. Determination of Aflatoxin G2 Content in Peanuts

The residual aflatoxin G2 content in peanut samples was determined using the post-column derivatization method. The mixture of acetonitrile methanol (1:1) solution was extracted, and the extract was purified and enriched by an immunoaffinity column. The purified solution was concentrated, volumetric, and filtered before being separated by liquid chromatography. Post-column derivatization (iodine reagent derivatization) was performed, detected by a fluorescence detector, and quantified by an external standard method.

Weigh 5 g of fully crushed sample into a 50 mL centrifuge tube, add 25 mL of 70% methanol aqueous solution, vortex, mix well, let stand at 7156× *g* for 5 min, and take the supernatant for backup. Transfer 5 mL of the supernatant into a 50 mL centrifuge tube, add 10 mL of ultrapure water, shake well, purify with an immunoaffinity column, mix well, and pass through an organic filter membrane. Collect the filtrate in an injection bottle for analysis.

The measurement conditions are as follows: mobile phase: A phase is water, B phase is acetonitrile methanol (1:1). The elution conditions of equal gradient are A 68%, B 32%, and C18 column with a column length of 150 mm, an inner diameter of 4.6 mm, and a filling material diameter of 5 μm. The flow rate is 1.0 mL/min, the column temperature is 40 °C, the single sample amount is 50 mL, the derivative solution is 0.05% iodine solution, the flow rate is 0.2 mL/min, and the temperature of the derivative reaction tube is 70 °C; The excitation wavelength of the fluorescence detector is 360 nm, and the emission wavelength is 440 nm.

### 5.11. Statistical Analysis

The peanuts and corn infected by colonies were measured by ImageJ 1.52a software, the data were analyzed by IBM SPSS Statistics 27, and the differential effect was analyzed by one-way analysis of variance (ANOVA). Using GraphPad Prism 8 and Adobe Illustrator CC 2019 drawing, various results were drawn on the Baimaike cloud platform based on the results of sample sequencing (http://www.biomarker.com.cn/biocloud/fenxi, accessed on 2 June 2022).

### 5.12. Data Submission

All the amplicon sequencing datasets in this study were submitted to the NCBI Sequence Read Archive (SRA) under accession number PRJNA1018336.

## Figures and Tables

**Table 1:** Determination of aflatoxin residues in peanuts by post-column derivatization chromatography.

Sample Name	Detection Result (ppb)
	AFG2
CK	3.4
100 μg/mL	0
200 μg/mL	0

## Data Availability

The datasets supporting the conclusions of this article are included within the article and its additional file. The datasets generated during and analyzed during the current study are available from the corresponding author upon reasonable request.

## Supplementary Materials

The following supporting information can be downloaded at: https://www.mdpi.com/article/10.3390/toxins15120667/s1, Figure S1: Correlation heat map between the control group and Myco treatment group; Table S1: Statistics of sequencing data; Table S2: Specific primer sequences used in RT-qPCR; Table S3: Fluorescence quantitative PCR reaction system; Table S4: Real-time PCR reaction program.
