# Supplementary material for: Mechanism Underlying Bacillus subtilis BS-Z15 Metabolite-Induced Prevention of Grain Contamination by Aspergillus flavus
Source: Toxins (Basel). 2023 Nov 22;15(12):667. doi: 10.3390/toxins15120667 (PMC10748098; doi:10.3390/toxins15120667)
Supplement: Supplementary file 1 [file toxins-15-00667-s001.zip › toxins-2678305-supplementary.pdf]

toxins

MDPI

Supplementary Materials: Mechanism Underlying *Bacillus subtilis* BS-Z15 Metabolite-Induced Prevention of Grain Contamination by *Aspergillus flavus*

Jingjing Zhao, Jun Yang, Haoran Li, Huanchen Ning, Jiayi Chen, Zhihui Chen, Heping Zhao and Huixin Zhao

Table S1. Statistics of sequencing data.

Samples	Clean reads	Clean bases	GC Content	%≥Q30
CK1	22,386,295	6,696,520,678	52.06%	94.39%
CK2	21,582,670	6,456,964,878	52.08%	94.21%
CK3	20,967,620	6,278,301,228	51.37%	94.19%
H201	20,606,012	6,171,415,484	52.45%	93.71%
H202	21,298,436	6,375,798,960	52.65%	93.79%
H203	22,341,048	6,689,538,538	52.56%	94.67%
H31	20,345,623	6,091,658,234	52.51%	94.48%
H32	20,661,009	6,187,646,738	52.60%	94.12%
H33	21,387,432	6,397,095,262	52.79%	94.18%
L201	21,148,316	6,332,071,044	52.59%	94.46%
L202	21,727,698	6,504,578,288	52.59%	94.30%
L203	20,762,266	6,217,574,048	52.29%	93.96%
L31	20,600,728	6,167,592,962	52.70%	94.14%
L32	21,167,586	6,334,672,286	52.72%	94.38%
L33	21,034,646	6,297,696,836	52.61%	94.21%

Note: H201-H203 is 20μg/mL 12h; H31-H33 is 20μg/mL 3h; L201-L203 is 4μg/mL 12h; L31-L33 is 4μg/mL 3h

Table S2. Specific primer sequences used in qRT-PCR.

Primer name	Primer sequences(5′-3′)
18S	F：GCTCTTTTGGGTCTCGTAATTGG
	R:CGCTATTGGAGCTGGAATTACC
aflR	F: CCTTTCTCACTACTCGGGTTT
	R:GCAGGTAATCAATAATGTCGG
aflT	F:GATTCTATTGCCTTGATTTTGG
	R:GGCGTAGTGCCCTGTCTTAT
aflP(omtA)	F:CACGCTTTCAGAGCAGGTAA
	R:TTCGGTGGAGGAGGGAGTT

Table S3. Fluorescence quantitative PCR reaction system.

Composition	50μL System
2xSuperReal PreMix Plus	25μL
50×ROX Reference Dye	1.0μL
Forward primers	1.5μL
Reverse Primers	1.5μL
RNA template	5μL
RNase-Free ddH_2O	Add to 50μL

Table S4. Real-time PCR reaction program.

Circulation	Stage	Temperature	Time	Contents
1×	Pre-mutability	95℃	15min	Pre-mutability
40×	PCR reactions	95℃	10sec	Gender reassignment
		50-60℃	20sec	Annealing
		72℃	30sec	Extension

1.00
0.80
0.60
0.40
0.20

	CK1	CK2	CK3	20 μg/mL 12h	20 μg/mL 12h	20 μg/mL 12h	20 μg/mL 3h	20 μg/mL 3h	20 μg/mL 3h	4 μg/mL 12h	4 μg/mL 12h	4 μg/mL 12h	4μg/mL 3h	4μg/mL 3h	4μg/mL 3h
4μg/mL 3h	0.577	0.587	0.541	0.463	0.519	0.564	0.903	0.923	0.930	0.536	0.550	0.519	0.938	0.986	1.000
4μg/mL 3h	0.651	0.660	0.578	0.402	0.463	0.505	0.931	0.944	0.964	0.480	0.495	0.466	0.972	1.000	0.986
4μg/mL 3h	0.666	0.671	0.529	0.337	0.406	0.452	0.950	0.958	0.980	0.423	0.438	0.420	1.000	0.972	0.938
4μg/mL 12h	0.103	0.112	0.126	0.963	0.984	0.973	0.338	0.389	0.390	0.986	0.988	1.000	0.420	0.466	0.519
4μg/mL 12h	0.111	0.121	0.136	0.972	0.996	0.978	0.351	0.404	0.408	0.999	1.000	0.988	0.438	0.495	0.550
4μg/mL 12h	0.100	0.111	0.126	0.974	0.996	0.973	0.333	0.386	0.391	1.000	0.999	0.986	0.423	0.480	0.536
20μg/mL 3h	0.699	0.693	0.573	0.311	0.379	0.434	0.975	0.986	1.000	0.391	0.408	0.390	0.980	0.964	0.930
20μg/mL 3h	0.650	0.640	0.547	0.304	0.374	0.440	0.991	1.000	0.986	0.386	0.404	0.389	0.958	0.944	0.923
20μg/mL 3h	0.675	0.664	0.558	0.258	0.321	0.386	1.000	0.991	0.975	0.333	0.351	0.338	0.950	0.931	0.903
20μg/mL 12h	0.112	0.118	0.137	0.948	0.980	1.000	0.386	0.440	0.434	0.973	0.978	0.973	0.452	0.505	0.564
20μg/mL 12h	0.090	0.100	0.115	0.978	1.000	0.980	0.321	0.374	0.379	0.996	0.996	0.984	0.406	0.463	0.519
20μg/mL 12h	0.071	0.081	0.108	1.000	0.978	0.948	0.258	0.304	0.311	0.974	0.972	0.963	0.337	0.402	0.463
CK3	0.815	0.813	1.000	0.108	0.115	0.137	0.558	0.547	0.573	0.126	0.136	0.126	0.529	0.578	0.541
CK2	0.994	1.000	0.813	0.081	0.100	0.118	0.664	0.640	0.693	0.111	0.121	0.112	0.671	0.660	0.587
CK1	1.000	0.994	0.815	0.071	0.090	0.112	0.675	0.650	0.699	0.100	0.111	0.103	0.666	0.651	0.577

Figure S1. Correlation heat map between the control group and Myco treatment group.
